# Postpartum depression risk prediction using explainable machine learning algorithms

**DOI:** 10.3389/fmed.2025.1565374

**Published:** 2025-08-07

**Authors:** Xudong Huang, Lifeng Zhang, Chenyang Zhang, Jing Li, Chenyang Li

**Affiliations:** ^1^Department of Science and Education, Shenyang Maternity and Child Health Hospital, Shenyang, China; ^2^Department of Maternal, Child and Adolescent Health, School of Public Health, Shenyang Medical College, Shenyang, China

**Keywords:** postpartum depression, machine learning, predictive model, influencing factors, maternal health

## Abstract

**Objective:**

Postpartum depression (PPD) is a common and serious mental health complication after childbirth, with potential negative consequences for both the mother and her infant. This study aimed to develop an explainable machine learning model to predict the risk of PPD and to identify its key predictive factors.

**Methods:**

A retrospective analysis was conducted on 1,065 women who attended their 6-week postpartum follow-up visit at a tertiary maternal and child healthcare hospital in Shenyang, China, from January to December 2021. Feature selection was performed using LASSO regression and the Boruta algorithm. Eight machine learning algorithms were then employed to construct the prediction models. Model performance was evaluated according to the area under the receiver operator characteristic curve (AUC), sensitivity, specificity, recall, F1 score, and accuracy. Shapley additive explanations (SHAP) were used to visualize the features of the model and individual case predictions.

**Results:**

Among the 1,065 women, 251 (23.5%) developed PPD. An 11-variable prediction model was developed, with XGBoost showing the best performance on both training and validation sets. After optimizing the model parameters and applying 10-fold cross-validation, the model achieved an average accuracy of 0.95, an average AUC of 0.955, average precision of 0.945, and average specificity of 0.985, indicating excellent predictive performance. The key predictive factors included weight gain during pregnancy, relationship with the mother-in-law, sleep quality, marital relationship, planned pregnancy, fetal sex preference, pregnancy-related anxiety, pelvic-floor muscle endurance, cervix status, attendance at prenatal education classes, and postpartum care satisfaction.

**Conclusion:**

The XGBoost model demonstrated optimal performance at predicting PPD and can aid healthcare professionals to identify high-risk individuals. The SHAP method enhanced the model’s interpretability, facilitating a better understanding of the causes of PPD, how to prevent it, and how to improve patient outcomes.

## 1 Introduction

Postpartum depression (PPD) is a prevalent mental disorder affecting women following childbirth (1). It is characterized by persistent depressive moods, emotional distress, and a notable loss of interest in daily activities. Epidemiological studies indicate that the prevalence of PPD varies widely across different regions, ranging from 5.0 to 26.32% (1–3). PPD has a profound impact on the physical and mental wellbeing of mothers, significantly diminishing their quality of life (2). It can have long-lasting adverse effects on the mother–infant relationship, as well as on the cognitive, emotional, and behavioral development of newborns (4). Therefore, it is crucial to identify high-risk mothers early and implement timely interventions to reduce the incidence of PPD and enhance both maternal and infant health.

In response to this growing global concern, mental healthcare for postpartum women has received increasing attention in recent years. International guidelines, such as those from the American College of Obstetricians and Gynecologists (ACOG), and Chinese national guidelines, emphasize the importance of routine screening for PPD during the perinatal period (5). In China, the standard of care for postpartum mental health typically includes routine screening for depressive symptoms during postnatal follow-up visits, such as the 6-week postpartum check-up (6). Recommended screening tools include standardized instruments such as the Edinburgh Postnatal Depression Scale (EPDS) and the Patient Health Questionnaire-9 (PHQ-9), which have been widely validated in both research and clinical practice (7, 8). Such screening aims to enable early detection and intervention, in line with tertiary prevention principles, to minimize the adverse effects of PPD on mothers and their infants. However, the practical implementation of these guidelines can be inconsistent, with variations in follow-up frequency and intervention strength across different regions and healthcare settings (9). Moreover, some perinatal women who develop PPD may be inclined to hide their clinical symptoms as a result of social stigma (10). Consequently, there is a pressing need for more effective and precise approaches to predict and prevent PPD.

Recent advancements in machine learning have demonstrated its powerful capability to process data and recognize patterns in the medical field and particularly to predict the risk of PPD (11, 12). Compared to traditional statistical methods such as logistic regression, machine learning algorithms can effectively handle high-dimensional data and complex non-linear relationships, uncovering hidden patterns that enhance predictive performance (13). However, current research remains in its nascent stages, with challenges such as small sample sizes and limited variable selection that affect the accuracy and clinical applicability of these models (14). Furthermore, existing studies rarely incorporate multidimensional risk factors, including postpartum physical examinations and pelvic-floor muscle function assessments, which further limit the clinical relevance of the models. While some studies have utilized machine learning methods for PPD prediction, there is a notable lack of comprehensive algorithm comparisons, parameter optimization, and exploration of model stability. Specifically, in Shenyang, China, models based on local data are absent, making it difficult to account for region-specific risk factors. To enhance the clinical utility of models, Shapley additive explanations (SHAP) could improve their interpretability, clarify the contribution of individual variables to the prediction, and increase model transparency and clinical operability (15). Therefore, developing a PPD risk prediction model based on comprehensive data and accurate algorithms remains a key focus of research.

This study aimed to construct a PPD risk prediction model using machine learning algorithms and incorporating multiple risk factors. We systematically compared the predictive performance of various algorithms to select the optimal model and facilitate its clinical application. The SHAP method was used to visually interpret the results of the model, to assist clinicians at identifying high-risk populations, and to implement timely interventions.

## 2 Methods

### 2.1 Sample selection

The participants in this study were women who visited a tertiary maternal and child healthcare hospital in Shenyang, China, for postpartum check-ups 6 weeks after giving birth, between January and December 2021. All participants underwent routine 6-week postpartum follow-up examinations conducted by trained physicians and nurses. During these examinations, clinical data were collected, including general health status, obstetric history, past medical history, and abdominal/pelvic dynamics. The follow-up assessments included a comprehensive health evaluation and adhered strictly to standardized clinical procedures to ensure data reliability and consistency. In addition, socio-psychological information was gathered through telephone follow-ups. Trained researchers conducted structured phone interviews with each participant to collect data on prenatal care, psychological education, and other psychosocial factors that might influence PPD. The telephone interviews were designed to ensure data accuracy and completeness. All collected data were entered into a database with strict confidentiality measures to safeguard participant privacy and data integrity.

The inclusion criteria for the study were as follows: (1) participants who returned for postpartum check-up at 6 weeks, (2) who underwent PPD screening, and (3) who volunteered to participate. Exclusion criteria included (1) women with pre-existing mental illnesses or severe organic diseases, such as heart, liver, or kidney conditions, and (2) participants with incomplete basic information. This study was approved by the Ethics Committee of Shenyang Maternal and Child Health Hospital (Approval No. 2023-017-01).

### 2.2 Research variables

A structured Chinese-language questionnaire was developed, informed by a literature review and expert consultations, to systematically collect data from postpartum women (16). Data collection involved two main sources: (1) clinical information obtained from standardized medical records completed by trained physicians and nurses during the 6-week postpartum visit, and (2) psychosocial data gathered through structured telephone interviews conducted by trained researchers.

Collected variables were grouped into four domains: demographic information, pregnancy and delivery variables, postpartum health conditions, and psychological and social factors, as detailed below. Demographic information included age, educational level, body mass index (BMI, kg/m^2^), family economic status, smoking and alcohol history, and pre-pregnancy menstrual cycle abnormalities (defined as menstrual cycles shorter than 21 days or longer than 35 days). Pregnancy and delivery variables encompassed parity, adverse obstetric history (including spontaneous abortion, induced abortion, termination of pregnancy, and ectopic pregnancy), gestational weight gain, pregnancy-related complications (e.g., thyroid dysfunction, gestational hypertension, and gestational diabetes), mode of delivery, perineal outcomes, instrumental delivery, manual placental removal, number of fetuses, preterm birth, abnormal birth weight (<2,500 g or ≥4,000 g), and adequacy of breast milk. Postpartum health conditions included self-reported pain, urinary and bowel dysfunction, and clinical assessments at the 6-week follow-up, including vaginal bleeding/discharge, abnormal pelvic or abdominal findings (including the vulva, vagina, cervix, uterus, and adnexa), hemorrhoids, and abdominal scars. Pelvic floor function was comprehensively evaluated, including measurements of muscle strength (Oxford grading 0–5), muscle endurance (0–5 s), tenderness, and dynamic pelvic pressure (cmH_2_O). Psychological and social factors—including perinatal sleep quality, fetal sex preference, participation in prenatal education, prenatal anxiety, satisfaction with postpartum confinement, marital relationship, and relationship with in-laws—were assessed via self-report during telephone interviews. A summary of the collected variables and their corresponding data sources is presented in Table 1.

**Table 1:** Overview of collected variables and data sources.

Domain	Variables	Data source
Demographic information	Age, body mass index (BMI, kg/m^2^), educational level, family economic status, smoking history, alcohol history, pre-pregnancy menstrual irregularities	Postpartum visit
Pregnancy and delivery variables	Parity, adverse obstetric history, gestational weight gain, pregnancy-related complications, mode of delivery, perineal outcomes, instrumental delivery, manual removal of placenta, number of fetuses, preterm birth, abnormal birth weight, adequacy of breast milk	Postpartum visit
Postpartum health	Vaginal bleeding/discharge, abnormal pelvic/abdominal findings (including the vulva, vagina, cervix, uterus, and adnexa), hemorrhoids, abdominal scars, pelvic floor muscle strength (Oxford scale), pelvic floor muscle endurance, pelvic floor tenderness, pelvic dynamic pressure (cmH₂O), postpartum pain, urinary dysfunction, bowel dysfunction	Postpartum visit
Psychological and social	Perinatal sleep quality, fetal sex preference, attendance at prenatal education, prenatal anxiety, satisfaction with postpartum confinement, marital relationship, in-law relationship	Telephone interview

### 2.3 Postpartum depression screening

The Chinese version of the EPDS was used to assess PPD. The EPDS is a widely used self-report tool for screening PPD. It consists of 10 items. Each item is scored based on the frequency of symptoms, ranging from 0 (not at all) to 3 (almost always), with a total score ranging from 0 to 30. A higher score indicates more severe depression. The Chinese version of the EPDS has been shown to have good internal consistency (Cronbach’s *α* = 0.714) and test–retest reliability (Cronbach’s *α* = 0.814) (17). In this study, a total score of ≥9 on the EPDS indicated PPD (18).

### 2.4 Statistical analysis and model construction

Data analysis was performed using DecisionLinnc 1.1.1.9, a platform that integrates multiple programming environments.^1^ It supports data processing, data analysis, and machine learning and offers an intuitive visual interface for conducting operations (19).

#### 2.4.1 Missing data imputation

To minimize the impact of missing data on model construction, variables with a missing rate of less than 20% were addressed using appropriate imputation methods (20). For continuous variables, the k-nearest neighbor algorithm was employed for imputation. Missing values were filled based on the characteristics of similar samples (21). For categorical variables, multiple imputation was applied, generating several imputed datasets and combining the results for subsequent analysis (20). Variables with a missing rate greater than 20% were excluded from the dataset.

#### 2.4.2 Statistical description

Continuous variables were tested for normality using the Kolmogorov–Smirnov test. As all continuous variables followed a non-normal distribution, they were described using the median (interquartile range) and compared between groups using the Mann–Whitney U test. Categorical variables were presented as percentages (%), and differences between groups were assessed using Pearson’s chi-squared test.

#### 2.4.3 Dataset splitting

When constructing the prediction model, the dataset was randomly split into a training set (comprising 70% of the total data) and a test set (comprising 30%). This commonly used 7:3 split aims to balance the need for sufficient training data with the necessity of evaluating model generalizability on unseen data (20). Such a ratio has been widely adopted in previous machine learning studies in healthcare domains (22, 23).

#### 2.4.4 Feature selection

Feature selection was performed on the training set using LASSO regression and the Boruta algorithm. LASSO regression, through L1 regularization, handles high-dimensional data, reduces multicollinearity, and selects significant features (24). A total of 100 lambda values were used, with the optimal parameter selected through 10-fold cross-validation, based on minimizing the cross-validation error (minimization rule). The Boruta algorithm is based on random forests. It evaluates the importance of features and further enhances the robustness of feature selection (15). In the Boruta feature-selection process, the confidence level was set to 0.01, the maximum number of iterations was set at 100, and the Bonferroni method was used to adjust the significance level for multiple comparisons. After independent feature selection using both methods, the intersection of the results was taken to ensure that the selected features exhibited greater stability and interpretability in the model.

#### 2.4.5 Prediction model construction and validation

To balance the distribution between PPD and non-PPD samples, and to mitigate the adverse impact of class imbalance on model training, we employed the synthetic minority oversampling technique (SMOTE) for oversampling the minority class (PPD samples). SMOTE generates synthetic samples by interpolating between minority-class samples, thereby increasing the number of PPD samples and improving sample quality (25). This prevents overfitting and enhances the model’s ability to recognize the PPD class, ultimately improving overall model performance.

Based on the variables selected through LASSO regression and the Boruta algorithm, eight machine learning models were constructed in the training set: a support vector machine (SVM), extreme gradient boosting (XGBoost), categorical boosting (CatBoost), naive Bayes (NB), random forests (RF), logistic regression, light gradient boosting machine (LightGBM), and adaptive boosting (AdaBoost). These models were designed to evaluate the performance of different algorithms in classification tasks. All models were constructed using the training set, and their performance was validated on an internal validation set. The predictive performance of the models was evaluated using several key metrics (26). The area under the receiver operating characteristic curve (AUC) assessed the model’s ability to distinguish between positive and negative samples. Sensitivity (recall) measured the proportion of correctly identified positive cases, and specificity evaluated the proportion of correctly identified negative cases. The F1 score, a harmonic mean of precision and recall, provided a balanced assessment, particularly for imbalanced datasets. Accuracy reflected the overall correctness of the model’s predictions. In addition, the Matthews correlation coefficient (MCC) was calculated to provide a reliable evaluation metric that accounts for true and false positives and negatives, offering a more informative and balanced assessment, especially when the dataset is imbalanced. Together, these metrics offered a comprehensive evaluation of the models’ performance.

To further optimize the models that performed best on both the training and test sets, a combination of grid search and 10-fold cross-validation was used to fine-tune their hyperparameters. After identifying the optimal hyperparameters, the models were retrained on the entire dataset using these optimal settings. Subsequently, 10-fold cross-validation was performed on the whole dataset to provide a more robust assessment of model performance. In addition, decision curve analysis (DCA) and precision–recall curves (PR curves) were plotted to demonstrate the real clinical utility of the models (27, 28). In DCA, the “treat all” strategy assumes that all patients are classified as high risk and would receive further psychological assessment or intervention, while the “treat none” strategy assumes no patients receive intervention. The net benefit of each model was compared with these two reference strategies across a range of threshold probabilities, providing an intuitive assessment of clinical value. The PR curve illustrates the trade-off between precision and recall across different thresholds, which is especially informative when dealing with imbalanced datasets.

#### 2.4.6 SHAP explainability analysis

**Figure 1:**
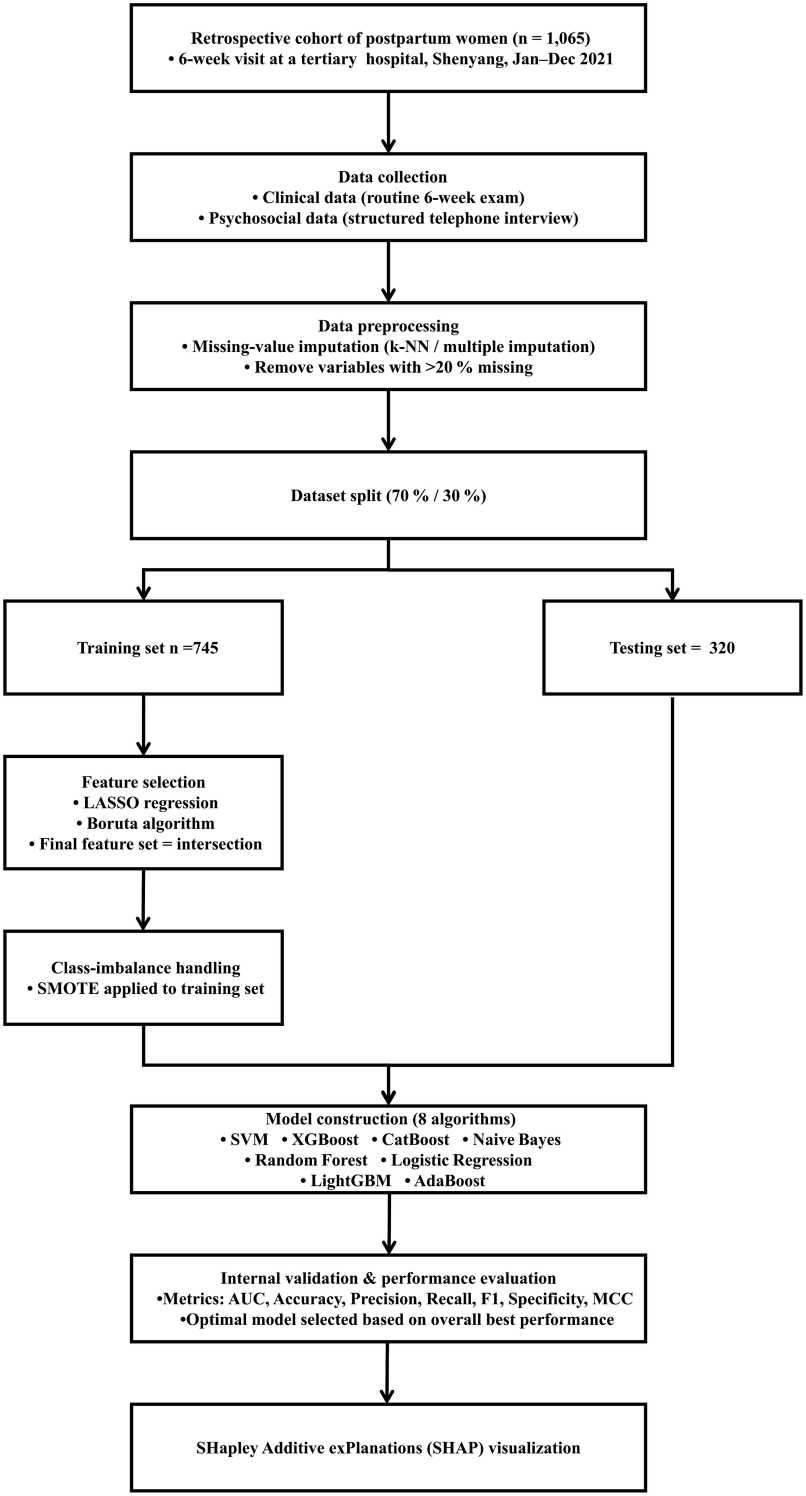
Analysis workflow for the development and evaluation of models. k-NN, k-nearest neighbors; SMOTE, synthetic minority oversampling technique; LASSO, least absolute shrinkage and selection operator; SVM, support vector machine; XGBoost, extreme gradient boosting; CatBoost, categorical boosting; NB, naive Bayes; RF, random forest; LightGBM, light gradient boosting machine; AUC, area under the receiver operating characteristic curve; MCC, Matthews correlation coefficient.

We employed the SHAP method to interpret the model outputs. SHAP is a game-theoretic technique that measures the impact of each input feature on the predictions of a model by assigning SHAP values (15). These values indicate the importance of each feature in the prediction process. SHAP values reflect the contribution of a feature to the prediction results, ensuring that the contributions from different features are fairly distributed. Specifically, a positive SHAP value indicates a positive influence on the prediction, and negative one signifies negative influence. Values close to zero suggest that the feature has a minimal contribution to the prediction result. We selected SHAP over other feature importance methods, such as Gini importance or permutation importance, because it provides consistent and locally accurate estimates, as well as both global and individual-level interpretability. This allows for clearer visualization and understanding of each feature’s impact on individual and overall predictions, which is essential for clinical decision support (29). To demonstrate the specific contributions of each feature to the prediction results, we used SHAP to generate bar charts, beeswarm plots, and force plots. Bar charts quantify the overall importance of each feature, beeswarm plots reveal the variation in feature impacts across different samples, and force plots illustrate the contribution of each feature for a specific sample. By evaluating the SHAP values of selected samples, we quantified the specific influence of each feature on the prediction, offering a deeper understanding of the model’s decision-making process. This analysis method not only highlights feature importance but also provides clear explanations for individual predictions, making the model’s decisions more transparent and easier to interpret. A summary of the entire methodological workflow is presented in Figure 1.

## 3 Results

### 3.1 Baseline characteristics

We collected information from 1,065 women at 6 weeks postpartum. The average age of participants was 29.66 years, and 251 women (23.5%) screened positive for PPD based on EPDS scores >9. The baseline characteristics are presented in Table 2. Significant group differences were observed across four domains. In terms of demographic information, women with PPD were slightly younger (*p* = 0.020), had lower educational attainment (*p* = 0.022), poorer economic status (*p* = 0.001), a higher prevalence of smoking (*p* < 0.001), and more frequent pre-pregnancy menstrual cycle abnormalities (*p* = 0.013). For pregnancy and delivery variables, those with PPD gained more weight during pregnancy (*p* = 0.011) and were more likely to report insufficient breast milk production (*p* = 0.008). Regarding postpartum health, higher rates of postpartum pain (*p* = 0.004) and urinary dysfunction (*p* = 0.019) were found in the PPD group. Finally, within the psychological and social domain, women with PPD were more likely to report fetal sex preference (*p* < 0.001), unplanned pregnancy (*p* < 0.001), no prenatal education (*p* = 0.041), poor perinatal sleep (*p* < 0.001), prenatal anxiety (*p* < 0.001), dissatisfaction with the postpartum experience (*p* = 0.002), and poor marital and in-law relationships (both *p* < 0.001).

**Table 2:** Comparison of baseline characteristics in the non-PPD and PPD groups.

Variables	Total (*N* = 1,065)	Non-PPD (*N* = 814)	PPD (*N* = 251)	*p*
Age (years), M (Q1, Q3)	29 (27–32)	29 (27–32)	29 (26–31)	0.020
Weight Gain During Pregnancy (Kg), M (Q1, Q3)	15 (11–20)	15 (11–19)	15 (12–20)	0.011
Body Mass Index (kg/m^2^), M (Q1, Q3)	24.038 (22.231–26.346)	24.093 (22.309–26.444)	23.855 (21.859–26.195)	0.384
Diastasis Recti (cm), M (Q1, Q3)	2 (1.5–2.5)	2 (1.5–2.5)	2 (1.5–2.5)	0.195
Pelvic Pressure (cmH_2_O), M (Q1, Q3)	63 (57–72)	63 (57–71)	63.2 (58–75)	0.552
Education Level(%), M (Q1, Q3)				
Below High School	93 (8.73)	65 (7.99)	28 (11.16)	0.022
Below Bachelor’s Degree	536 (50.33)	398 (48.89)	138 (54.98)	
Bachelor’s Degree or Above	436 (40.94)	351 (43.12)	85 (33.86)	
Economic Level (%)				
Poor	432 (40.56)	316 (38.82)	116 (46.22)	0.001
Moderate	516 (48.45)	419 (51.47)	97 (38.65)	
Good	117 (10.99)	79 (9.71)	38 (15.14)	
Smoke (%)				
Yes	125 (11.74)	79 (9.71)	46 (18.33)	<0.001
No	940 (88.26)	735 (90.29)	205 (81.67)	
Drink (%)				
Yes	78 (7.32)	58 (7.13)	20 (7.97)	0.757
No	987 (92.68)	756 (92.87)	231 (92.03)	
Preterm birth (%)				
Yes	73 (6.85)	51 (6.27)	22 (8.76)	0.220
No	992 (93.15)	763 (93.73)	229 (91.24)	
Number of births (%)				
1	1,055 (99.06)	807 (99.14)	248 (98.80)	0.915
2	10 (0.94)	7 (0.86)	3 (1.20)	
Cesarean section (%)				
Yes	448 (42.07)	336 (41.28)	112 (44.62)	0.387
No	617 (57.93)	478 (58.72)	139 (55.38)	
Painless delivery (%)				
Yes	551 (51.74)	421 (51.72)	130 (51.79)	1.000
No	514 (48.26)	393 (48.28)	121 (48.21)	
Episiotomy (%)				
Yes	192 (18.03)	145 (17.81)	47 (18.73)	0.814
No	873 (81.97)	669 (82.19)	204 (81.27)	
Perineal laceration (%)				
Yes	453 (42.54)	345 (42.38)	108 (43.03)	0.914
No	612 (57.46)	469 (57.62)	143 (56.97)	
Forceps delivery (%)				
Yes	32 (3.00)	23 (2.83)	9 (3.59)	0.685
No	1,033 (97.00)	791 (97.17)	242 (96.41)	
Manual removal of placenta (%)				
Yes	158 (14.84)	118 (14.50)	40 (15.94)	0.646
No	907 (85.16)	696 (85.50)	211 (84.06)	
Vaginal bleeding (%)				
Yes	216 (20.28)	162 (19.90)	54 (21.51)	0.642
No	849 (79.72)	652 (80.10)	197 (78.49)	
Postpartum pain (%)				
Yes	222 (20.85)	153 (18.80)	69 (27.49)	0.004
No	843 (79.15)	661 (81.20)	182 (72.51)	
Urinary dysfunction (%)				
Yes	157 (14.74)	108 (13.27)	49 (19.52)	0.019
No	908 (85.26)	706 (86.73)	202 (80.48)	
Bowel dysfunction (%)				
Yes	166 (15.59)	122 (14.99)	44 (17.53)	0.384
No	899 (84.41)	692 (85.01)	207 (82.47)	
Thyroid abnormalities during pregnancy (%)				
Yes	44 (4.13)	35 (4.30)	9 (3.59)	0.752
No	1,021 (95.87)	779 (95.70)	242 (96.41)	
Pregnancy induced hypertension (%)				
Yes	29 (2.72)	23 (2.83)	6 (2.39)	0.882
No	1,036 (97.28)	791 (97.17)	245 (97.61)	
Gestational diabetes (%)				
Yes	169 (15.87)	131 (16.09)	38 (15.14)	0.793
No	896 (84.13)	683 (83.91)	213 (84.86)	
Pregnancy complications (%)				
Yes	242 (22.72)	189 (23.22)	53 (21.12)	0.543
No	823 (77.28)	625 (76.78)	198 (78.88)	
Fetal weight abnormality (%)				
Yes	409 (38.40)	304 (37.35)	105 (41.83)	0.229
No	656 (61.60)	510 (62.65)	146 (58.17)	
Pre-pregnancy menstrual cycle abnormalities (%)				
Yes	132 (12.39)	89 (10.93)	43 (17.13)	0.013
No	933 (87.61)	725 (89.07)	208 (82.87)	
Primipara (%)				
Yes	866 (81.31)	657 (80.71)	209 (83.27)	0.415
No	199 (18.69)	157 (19.29)	42 (16.73)	
Adverse obstetric history (%)				
Yes	337 (31.64)	246 (30.22)	91 (36.25)	0.086
No	728 (68.36)	568 (69.78)	160 (63.75)	
Adequate breast milk (%)				
Yes	684 (64.23)	541 (66.46)	143 (56.97)	0.008
No	381 (35.77)	273 (33.54)	108 (43.03)	
Feeding method (%)				
Breastfeeding	897 (84.23)	685 (84.15)	212 (84.46)	0.985
Mixed Feeding	168 (15.77)	129 (15.85)	39 (15.54)	
Abdominal scar (%)				
Yes	422 (39.62)	312 (38.33)	110 (43.82)	0.138
No	643 (60.38)	502 (61.67)	141 (56.18)	
Pubic symphysis pain (%)				
Yes	265 (24.88)	190 (23.34)	75 (29.88)	0.044
No	800 (75.12)	624 (76.66)	176 (70.12)	
Vulva (%)				
Normal	624 (58.59)	476 (58.48)	148 (58.96)	0.949
Abnormal	441 (41.41)	338 (41.52)	103 (41.04)	
Vagina (%)				
Normal	835 (78.40)	638 (78.38)	197 (78.49)	1.000
Abnormal	230 (21.60)	176 (21.62)	54 (21.51)	
Cervix (%)				
Normal	916 (86.01)	704 (86.49)	212 (84.46)	0.481
Abnormal	149 (13.99)	110 (13.51)	39 (15.54)	
Uterus (%)				
Normal	1,037 (97.37)	792 (97.30)	245 (97.61)	0.964
Abnormal	28 (2.63)	22 (2.70)	6 (2.39)	
Adnexa (%)				
Normal	1,033 (97.00)	789 (96.93)	244 (97.21)	0.986
Abnormal	32 (3.00)	25 (3.07)	7 (2.79)	
Hemorrhoids (%)				
Yes	453 (42.54)	341 (41.89)	112 (44.62)	0.489
No	612 (57.46)	473 (58.11)	139 (55.38)	
Pelvic floor tenderness (%)				
Yes	89 (8.36)	67 (8.23)	22 (8.76)	0.891
No	976 (91.64)	747 (91.77)	229 (91.24)	
Pelvic floor muscle strength (%)				
0	22 (2.07)	19 (2.33)	3 (1.20)	0.180
1	381 (35.77)	305 (37.47)	76 (30.28)	
2	355 (33.33)	258 (31.70)	97 (38.65)	
3	206 (19.34)	155 (19.04)	51 (20.32)	
4	78 (7.32)	58 (7.13)	20 (7.97)	
5	23 (2.16)	19 (2.33)	4 (1.59)	
Pelvic floor muscle endurance (%)				
0	78 (7.32)	65 (7.99)	13 (5.18)	0.363
1	498 (46.76)	387 (47.54)	111 (44.22)	
2	285 (26.76)	215 (26.41)	70 (27.89)	
3	135 (12.68)	97 (11.92)	38 (15.14)	
4	50 (4.69)	35 (4.30)	15 (5.98)	
5	19 (1.78)	15 (1.84)	4 (1.59)	
Type I pelvic floor muscles (%)				
0	315 (29.58)	244 (29.98)	71 (28.29)	0.956
1	273 (25.63)	209 (25.68)	64 (25.50)	
2	131 (12.30)	100 (12.29)	31 (12.35)	
3	80 (7.51)	62 (7.62)	18 (7.17)	
4	34 (3.19)	27 (3.32)	7 (2.79)	
5	232 (21.78)	172 (21.13)	60 (23.90)	
Type II pelvic floor muscles (%)				
0	212 (19.91)	160 (19.66)	52 (20.72)	0.914
1	144 (13.52)	113 (13.88)	31 (12.35)	
2	123 (11.55)	98 (12.04)	25 (9.96)	
3	99 (9.30)	75 (9.21)	24 (9.56)	
4	74 (6.95)	57 (7.00)	17 (6.77)	
5	413 (38.78)	311 (38.21)	102 (40.64)	
Fetal sex preference (%)				
Yes	287 (26.95)	196 (24.08)	91 (36.25)	<0.001
No	778 (73.05)	618 (75.92)	160 (63.75)	
Planned pregnancy (%)				
Yes	745 (69.95)	598 (73.46)	147 (58.57)	<0.001
No	320 (30.05)	216 (26.54)	104 (41.43)	
Prenatal education class (%)				
Yes	404 (37.93)	322 (39.68)	81 (32.27)	0.041
No	661 (62.07)	491 (60.32)	170 (67.73)	
Perinatal sleep status (%)				
Good	626 (58.78)	510 (62.65)	116 (46.22)	<0.001
Average	268 (25.16)	223 (27.40)	45 (17.93)	
Poor	171 (16.06)	81 (9.95)	90 (35.86)	
Prenatal anxiety (%)				
Yes	217 (20.38)	116 (14.25)	101 (40.24)	<0.001
No	848 (79.62)	698 (85.75)	150 (59.76)	
Satisfaction With postpartum confinement (%)				
Satisfied	762 (71.55)	602 (73.96)	160 (63.75)	0.002
Unsatisfied	303 (28.45)	212 (26.04)	91 (36.25)	
Marital relationship (%)				
Good	955 (89.67)	759 (93.24)	196 (78.09)	<0.001
Poor	110 (10.33)	55 (6.76)	55 (21.91)	
In law relationship (%)				
Good	879 (82.54)	708 (86.98)	171 (68.13)	<0.001
Poor	186 (17.46)	106 (13.02)	80 (31.87)	

PPD, postpartum depression; M, median; Q1, 1st quartile; Q3, 3rd quartile; pregnancy complications, including thyroid disorders, gestational hypertension, and gestational diabetes; adverse obstetric history, including spontaneous abortion, induced abortion, termination of pregnancy, and ectopic pregnancy.

The samples were randomly split into a training group (745 cases) and a testing group (320 cases) in a 7:3 ratio. In the training set, the non-PPD group accounted for 76.78% (*n* = 572) and the PPD group for 23.22% (*n* = 173), while in the testing set, the non-PPD group comprised 75.62% (*n* = 242) and the PPD group 24.38% (*n* = 78). There was no significant difference in the prevalence of PPD between the training and testing sets (*p* = 0.684). However, significant differences between the two sets were observed in preterm birth, painless delivery, forceps delivery, and gestational diabetes (all *p* < 0.05), while no significant differences were found in the other variables. This indicates that the baseline characteristics of the training and testing sets were generally balanced and comparable. Details of the group differences are provided in Supplementary Table 1.

### 3.2 Feature selection

**Figure 2:**
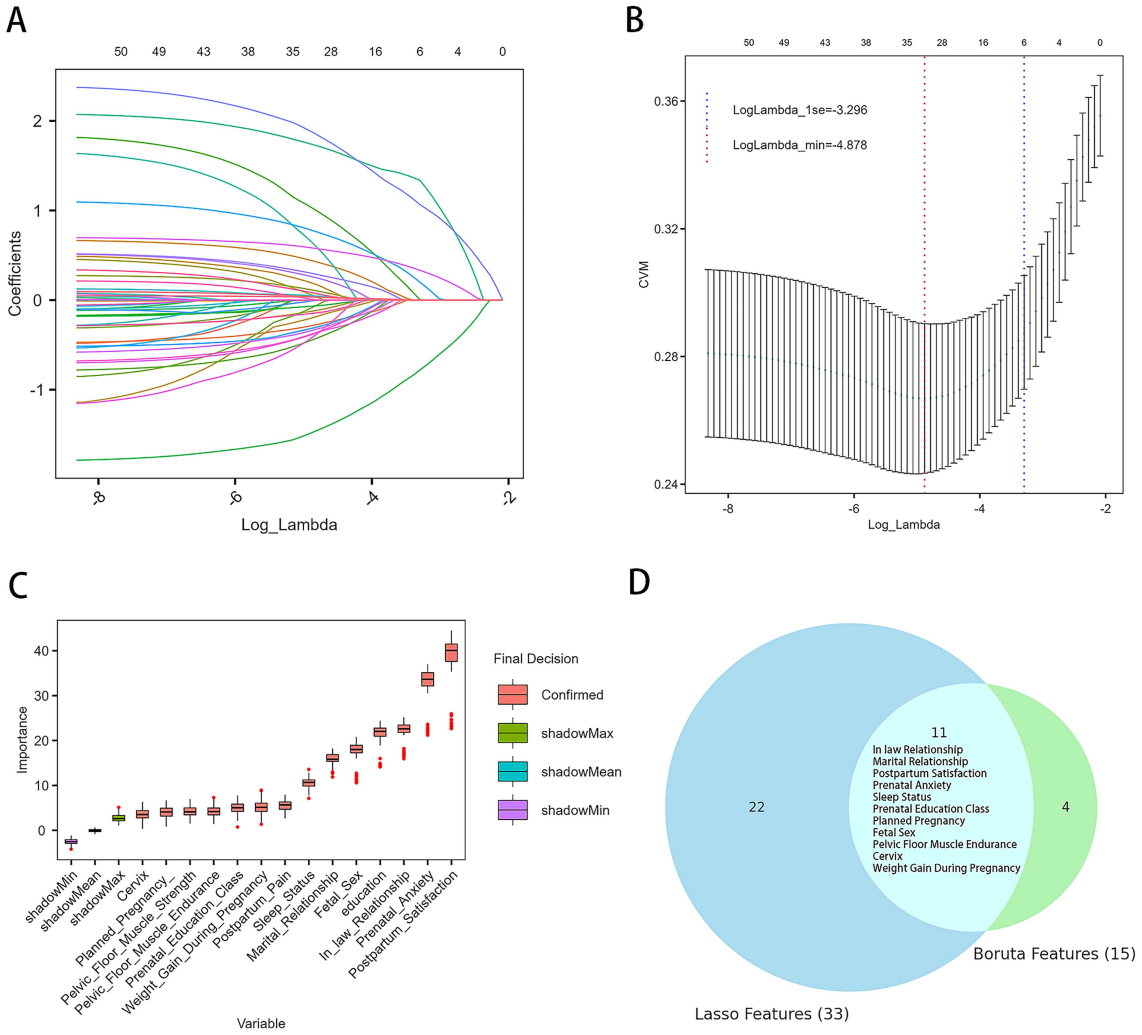
Results of feature selection. **(A)** Trajectory of variables selected by the LASSO regression model. **(B)** Ten-fold cross-validation curve for lambda selection; the vertical axis represents the cross-validation mean (CVM), that is, the mean cross-validation error for each lambda value. The left dashed line marks the optimal lambda value (lambda.min), and the right dashed line marks the lambda value within one standard error of the minimum (lambda.1se). **(C)** Results from the Boruta algorithm for feature selection. Red boxplots represent features confirmed as important. Green, blue, and purple boxplots indicate the distributions of shadow features used as the baseline for comparison. Features with importance significantly higher than the best shadow were retained for model development. **(D)** Common predictive variables selected by both Boruta and LASSO.

First, LASSO regression was performed on the variables in the training set, resulting in the identification of 33 variables associated with PPD at lambda.min = −4.876 (Figures 2A,B). Then, Boruta feature selection was conducted with a confidence level of 0.01, a maximum of 100 iterations, and Bonferroni adjustment for multiple comparisons. Figure 2C displays the results, in which the importance of each original feature is compared with that of randomly generated shadow features (green, blue, and purple boxplots). Features marked in red (“confirmed”) demonstrated significantly higher importance than the shadow features and were therefore retained, resulting in 15 important predictors for subsequent model development. Finally, by cross-referencing the features selected by both Boruta and LASSO regression, a common subset of 11 predictive features was identified (Figure 2D): weight gain during pregnancy, mother-in-law relationship, sleep quality, marital relationship, planned pregnancy, fetal sex preference, pregnancy-related anxiety, pelvic-floor muscle endurance, cervix condition, satisfaction with postpartum confinement, and participation in prenatal education. These features were used for the subsequent model construction.

### 3.3 Model construction and performance comparison

After completing the feature selection process, the SMOTE algorithm was applied to balance the data in the training set to address the issue of data imbalance. Following this, the final training set consisted of 1,162 cases: 581 from the PPD group and 581 from the non-PPD group.

**Figure 3:**
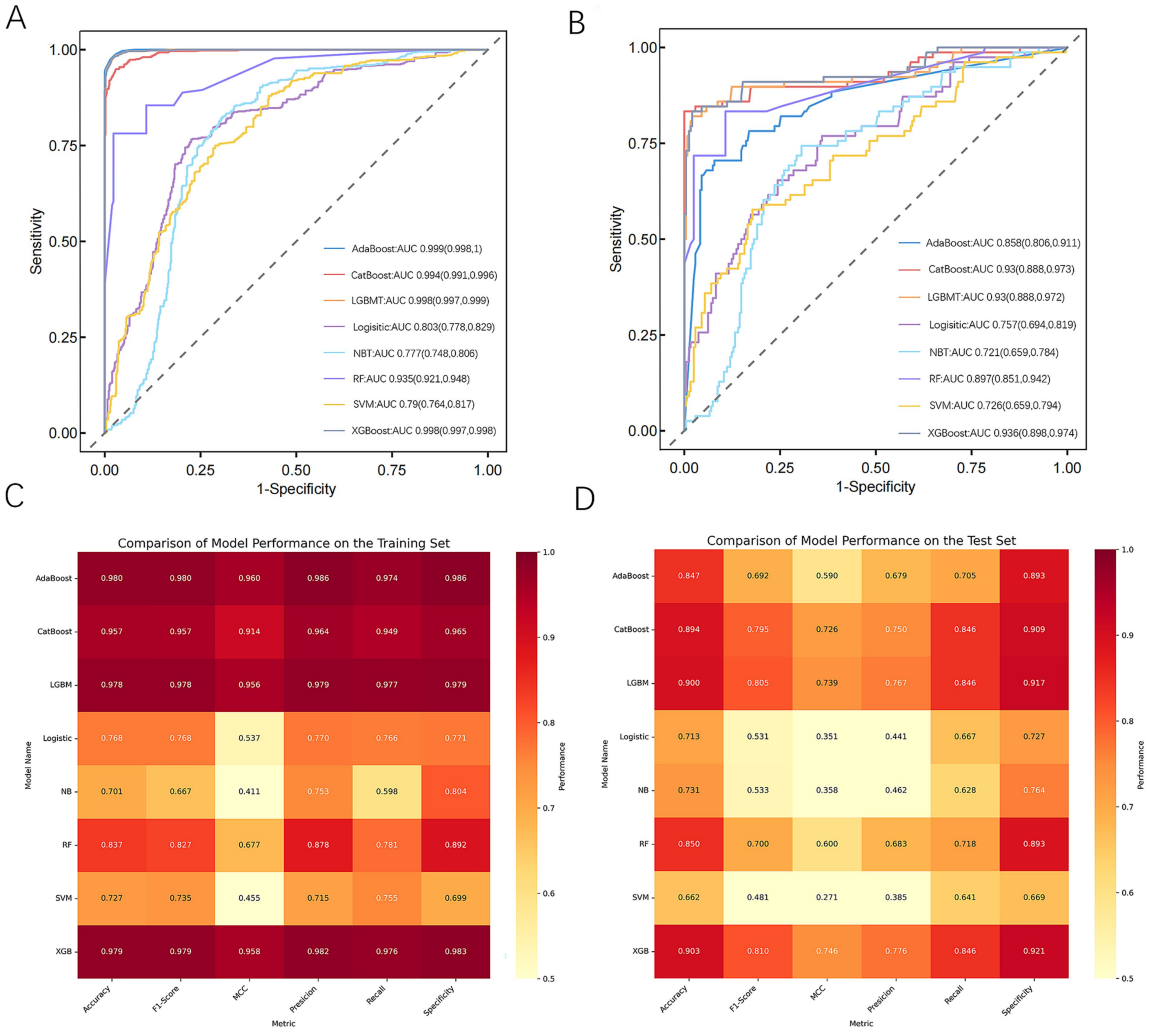
Performance and comparison of predictive models. **(A)** ROC curve for the training dataset. **(B)** ROC curve for the test dataset. **(C)** Evaluation metrics for the training dataset. **(D)** Evaluation metrics for the test dataset. AdaBoost, adaptive boosting; CatBoost, categorical boosting; LightGBM, light gradient boosting machine; Logistic, logistic regression; NB, naive Bayes; RF, random forest; SVM, support vector machine; XGBoost, extreme gradient boosting; MCC, Matthews correlation coefficient.

Eight machine learning models were built to identify the risk of PPD in mothers 6 weeks after giving birth. The performance of each model on the training set was evaluated using accuracy, recall, F1 score, MCC, specificity, and AUC (Figures 3A, C). The results showed that all models exhibited high performance at predicting PPD, with AdaBoost, XGBoost, and LightGBM achieving the best results. Their accuracy rates were 0.98, 0.979, and 0.978, respectively, and they demonstrated excellent performance in key metrics such as the AUC and precision, indicating strong discrimination ability between positive and negative samples. CatBoost and RF also performed well, with accuracies of 0.957 and 0.837, respectively. By contrast, NB and SVM performed poorly across all metrics, especially NB, which had a high false-negative rate of 40%, limiting its applicability.

To identify the optimal model, all models were further validated on the test set, with results presented in Figures 3B, D. The results showed that CatBoost, XGBoost, and LGBM all maintained stable and comparable performance on the test set, with AUCs of 0.93, 0.936, and 0.93, respectively, demonstrating good generalization ability. On the other hand, AdaBoost performed excellently on the training set, with an AUC of only 0.858 on the test set, indicating potential overfitting.

### 3.4 Hyperparameter optimization and validation of the optimal model

**Figure 4:**
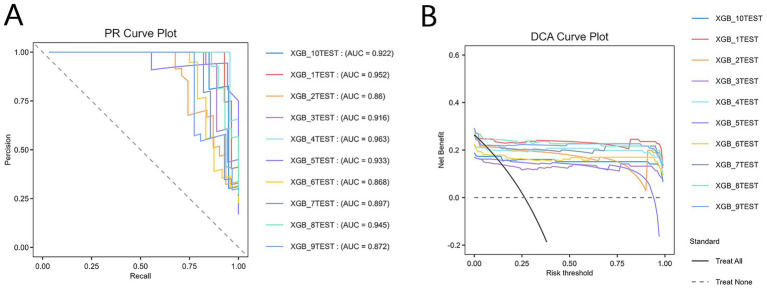
Comprehensive evaluation of the XGBoost model. **(A)** Precision–recall (PR) curve. **(B)** Decision curve analysis (DCA) curve. XGB, extreme gradient boosting. The “treat all” curve represents the benefit rates for all cases with intervention, while the “treat none” curve represents the benefit rates for all cases without intervention.

Given the strong generalization ability, balanced accuracy and recall, high specificity, and relatively stable performance of the XGBoost model on both the training and test sets, it was selected as the optimal model. Hyperparameter optimization was performed using a combination of grid search and 10-fold cross-validation. The final XGBoost model, constructed based on the optimal parameters, was evaluated through 10-fold cross-validation. The results revealed an average accuracy of 0.95, average AUC of 0.955, average precision of 0.945, and average specificity of 0.985, demonstrating superior performance. Furthermore, the PR curve (Figure 4A) and DCA curve (Figure 4B) generated through 10-fold cross-validation exhibited favorable net benefits across a range of thresholds, confirming the model’s robustness and clinical utility. The PR curve (Figure 4A) revealed that the model achieved robust precision and recall across various thresholds, reflecting strong discriminative ability in identifying women at risk for PPD. In the DCA plot, the “treat all” strategy represents a hypothetical scenario where all postpartum women are assumed to be at high risk and thus receive intervention (such as psychological evaluation or preventive counseling), while the “treat none” strategy corresponds to no intervention for any women. The net benefit of our prediction model consistently exceeded both “treat all” and “treat none” strategies across multiple thresholds, indicating superior clinical utility in identifying women most likely to benefit from targeted intervention.

### 3.5 SHAP-based model interpretability analysis

**Figure 5:**
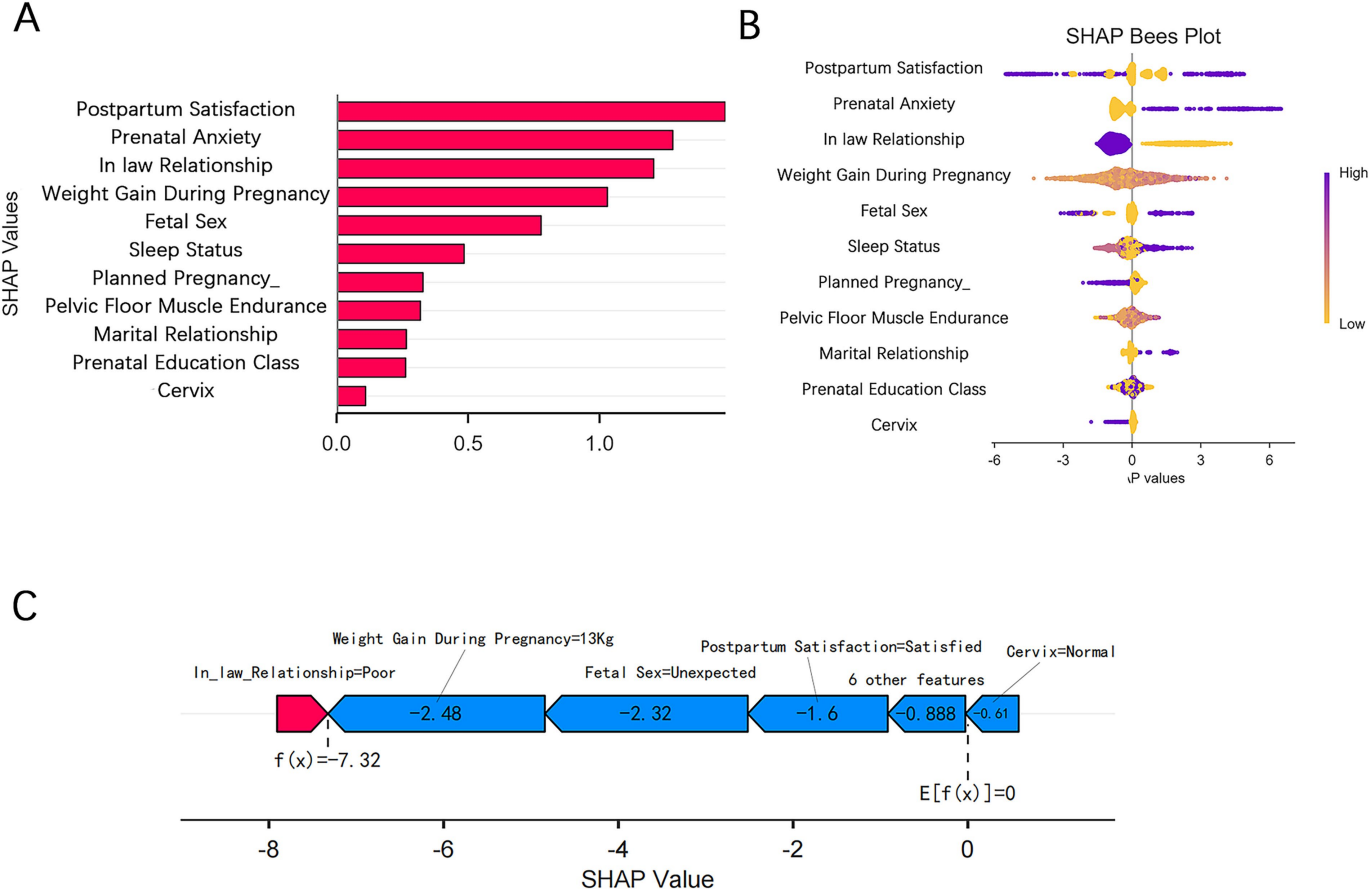
SHAP visualizations for interpreting the machine learning model. **(A)** SHAP bar plot. **(B)** SHAP beeswarm plot. **(C)** SHAP force plot.

We evaluated the relative importance of various factors influencing the susceptibility to PPD in women. Figure 5A presents the feature importance ranking in the XGBoost model, with the vertical axis ordered by descending importance, and the horizontal axis representing the average SHAP values. The analysis identified five key factors affecting PPD: satisfaction with postpartum confinement, prenatal anxiety, mother-in-law relationship, weight gain during pregnancy, and whether the fetal sex met expectations. Figure 5B illustrates the SHAP values for each feature in the XGBoost model, where the horizontal axis shows the SHAP values and the vertical axis ranks the features based on their cumulative SHAP values. Each data point corresponds to an instance, with the X-axis indicating the SHAP value of the corresponding feature. To provide a clearer understanding of the model’s decision-making process, we performed a detailed analysis on a representative sample, as shown in the figure. Figure 5C demonstrates the prediction process for this sample, with red indicating a positive contribution, blue representing a negative impact, and the f(x) value corresponding to the SHAP value for each factor.

## 4 Discussion

### 4.1 Postpartum depression screening positive rate

PPD is a common complication in women, posing significant risks to both maternal and neonatal health and bringing substantial social and economic burdens. In this study, the overall positive screening rate for depression in women at 6 weeks postpartum was 23.57% (251/1065), which is higher than the global reported rate of 14% (95% CI: 12.0–15.0%) and the rate of 21.4% (95% CI: 15.2–27.6%) reported in China (1, 17). These differences may be attributed to factors such as the different screening tools used, the timing of the screenings, the standards applied, regional variations, and sample sizes across studies (1, 17). Although these differences may be influenced by cultural, economic, and lifestyle factors, as well as selection bias inherent in this study, they still underscore the importance of addressing PPD. This highlights the urgent need for clinical prevention and treatment efforts to reduce the incidence and mitigate the harmful effects of PPD, ultimately promoting maternal mental health.

### 4.2 Postpartum depression risk predictors and interpretability analysis

We used postpartum follow-up data at 6 weeks, specifically incorporating gynecological assessments (such as pelvic-floor muscle function tests), to provide a comprehensive clinical context for the PPD prediction model. Given that the data were derived from routine postpartum checks at 6 weeks, the model benefits from high practicality and clinical feasibility. By combining the Boruta algorithm and LASSO regression, we accurately identified 11 key features associated with PPD: gestational weight gain, mother-in-law relationship, sleep quality, marital relationship, planned pregnancy, fetal sex preference, prenatal anxiety, pelvic-floor muscle endurance, cervix status, prenatal education class, and postpartum satisfaction. These findings are consistent with those from previous studies, further validating the relevance of these factors for predicting PPD (1, 17, 30).

To enhance the clinical applicability of the model, we utilized the SHAP method for both global and local explanations, clearly identifying the contribution of each predictor to the model’s decision-making process. The SHAP values revealed that satisfaction with postpartum confinement, prenatal anxiety, mother-in-law relationship, gestational weight gain, and fetal sex preference were the top five features. Satisfaction with postpartum confinement reflects the mother’s psychological wellbeing, and inadequate support during this period may increase the risk of depression (31–33). Prenatal anxiety often persists postpartum, exacerbating depressive tendencies (30). The relationship with the mother-in-law influences the maternal family support system, and a strained relationship significantly increases emotional distress (16, 34). Gestational weight gain may elevate depression risk through mechanisms such as inflammation pathways and weight-related anxiety (35, 36). Finally, in East Asian cultures, a mismatch between expected and actual fetal sex may trigger feelings of disappointment, thereby increasing the likelihood of depression (1, 37).

### 4.3 Performance of postpartum depression risk prediction model

Eight machine learning algorithms were employed in our study to construct a PPD prediction model based on 11 clinical variables collected at 6 weeks postpartum. The results demonstrated that the XGBoost algorithm performed exceptionally well, exhibiting strong discriminative power and calibration ability. In addition, it showed significant net benefits in clinical practice. The stability and accuracy of the model were further confirmed by performing 10-fold cross-validation on the entire dataset.

In recent years, machine learning has been widely applied to predict the risk of PPD. Zhang et al. achieved high accuracy using a random forests algorithm (14). However, their model lacked interpretability as it did not clearly identify which features were most influential in predicting PPD. This “black-box” nature makes it difficult for clinicians to understand or trust the model’s predictions, thereby limiting its clinical usefulness. Moreover, many studies directly exclude missing data samples, which could lead to sample selection bias (44). In our study, for variables with a missing rate less than 20%, missing data were handled using a combination of multiple imputation and k-nearest neighbors, enhancing the accuracy, reliability, and generalizability of the predictive model while avoiding selection and prediction biases (20, 25). Compared with traditional methods, the improvements in this study ensured greater model stability and better predictive performance (38). Furthermore, in PPD clinical research, imbalanced datasets are common, and traditional statistical methods have limited effectiveness at handling class imbalance (11, 12). In recent years, improved sampling methods and classification algorithms have been increasingly applied to address this issue (25). Unlike traditional oversampling methods that duplicate samples, the SMOTE algorithm generates new samples of the minority class by creating synthetic neighbors, thus avoiding data inflation and increased training complexity (15). In our study, we used the SMOTE algorithm to balance the dataset by increasing the number of minority-class samples, improving inter-group comparability. The results demonstrated that SMOTE significantly enhanced model performance, improved minority-class identification, and thus improved overall classification accuracy.

XGBoost is an efficient algorithm based on the gradient boosting tree framework, known for its ability to handle large-scale datasets, missing values, and efficient parallel computation (39, 40). It is particularly suitable for complex feature spaces and non-linear problems. In recent years, XGBoost has been widely applied in the medical field, demonstrating excellent performance in areas such as sepsis, cardiovascular diseases, and renal injury (39, 41, 42). Compared to traditional logistic regression, XGBoost constructs models by integrating multiple weak classifiers, allowing it to capture non-linear relationships and enhance generalization (40). In addition, it exhibits strong robustness to outliers and noisy data. Hochman et al. analyzed Israel’s electronic health records database and built a predictive model based on XGBoost to assess the risk of developing PPD within 1 year (43). The model achieved an AUC of 0.712 (95% CI: 0.690–0.733), indicating moderate predictive ability. Moreover, the XGBoost algorithm can automatically interpret interactions among independent variables and handle missing data in decision tree branches, thereby improving model performance. Our study further validated the effectiveness of XGBoost for PPD prediction. However, model performance may vary depending on factors such as the population, variable selection, and parameter tuning. Therefore, it is essential to select an appropriate algorithm based on experimental needs and data characteristics and to ensure model performance and interpretability after a thorough exploration and evaluation of the data.

### 4.4 Limitations

The retrospective design of our study renders it subject to confounding factors and selection bias, which could limit the validity and generalizability of the results. The relevant variables were retrospectively collected, and information bias may exist. In addition, the study was conducted at a single center with a relatively small sample size, which affects the robustness and applicability of the findings. Future multi-center studies are needed to enhance the generalizability of the model across different settings. Although this study incorporated a wide range of clinical and demographic variables, some potential confounders such as biomarkers, genetics, lifestyle, and environmental factors may have been overlooked. This could affect the accuracy of the results. In addition, several predictors in our model—such as fetal sex preference, satisfaction with postpartum confinement, and mother-in-law relationship—are culturally specific and may not generalize well to populations in other sociocultural contexts. The study relied on screening scales for diagnosis, lacking confirmatory diagnostic assessments, which may reduce diagnostic precision. Furthermore, the study only assessed risk factors at 6 weeks postpartum and did not involve long-term follow-ups, limiting the ability to capture the dynamic changes in depressive symptoms. Finally, internal validation was performed only on the development dataset, and no external validation was conducted. Despite these limitations, our findings provide important insights into the use of interpretable machine learning models for PPD risk prediction, underscoring their potential to improve early identification and targeted intervention in clinical practice. Further validation in larger and more diverse populations will be essential to confirm these results and facilitate clinical implementation.

### 4.5 Clinical implications and application potential

Building upon the demonstrated strengths of XGBoost, our study assessed its applicability in predicting PPD using clinical and psychosocial data obtained during routine 6-week postpartum visits. The model achieved favorable predictive performance in our dataset, suggesting its potential as a supportive tool to assist in identifying women at elevated risk of PPD. Importantly, the identification of key modifiable risk factors also provides an opportunity for early, targeted intervention during the perinatal period. For example, modifiable factors such as poor sleep quality, prenatal anxiety, strained marital or mother-in-law relationships, and low satisfaction with postpartum care could potentially be addressed through targeted counseling, psychoeducation, or strengthened perinatal support services. When used judiciously, the model may aid in tailoring preventive strategies and optimizing screening efforts, particularly in settings where mental health resources are limited. Nevertheless, these findings should be interpreted with caution, and further validation in larger, multi-center cohorts is warranted to ensure broader applicability.

To enhance clinical integration, the model could be deployed through web-based calculators, mobile applications, or embedded within electronic health record systems. However, practical implementation requires addressing interoperability with existing hospital systems and ensuring that healthcare providers receive adequate support to interpret machine learning-derived outputs, including SHAP-based explanations. Moreover, potential misclassification—such as false positives or false negatives—carries clinical and psychological implications. These considerations highlight the importance of using model predictions to complement, rather than replace, clinical judgment and longitudinal symptom monitoring. Efforts should also be made to minimize the potential psychological impact of risk labeling and ensure appropriate communication and counseling are provided when needed.

## 5 Conclusion

In this study, we developed an XGBoost model to predict the risk of PPD in women 6 weeks after delivery. Our findings suggest that the XGBoost model holds potential as a clinically useful tool, with enhanced interpretability through SHAP values, which help clinicians better understand relevant risk factors. However, the single-center nature of this study and the lack of biological variables may limit generalizability. Further prospective, multi-center validation is required before clinical implementation.

## Data Availability

The raw data supporting the conclusions of this article will be made available by the authors, without undue reservation.

## Footnotes

^1^ https://www.statsape.com/
