# Supplementary material for: Postpartum depression risk prediction using explainable machine learning algorithms
Source: Front Med (Lausanne). 2025 Aug 7;12:1565374. doi: 10.3389/fmed.2025.1565374 (PMC12367785; doi:10.3389/fmed.2025.1565374)
Supplement: Supplementary file 1 [file Table_1.docx]

**Supplementary Table 1.** Comparison of Baseline Characteristics Between the Training and Testing Sets

| Variables | Total(N=1065) | training group  (n = 745) | testing group  (n = 320) | statistic | *P* |
| --- | --- | --- | --- | --- | --- |
|  |  |  |  |  |  |
| Age (years) ,M (Q1, Q3) | 29.00 (27.00, 32.00) | 29.00 (27.00, 32.00) | 29.00 (27.00, 32.00) | Z=-0.13 | 0.899 |
| Weight Gain During Pregnancy (Kg) ,M (Q1, Q3) | 15.00 (11.00, 20.00) | 15.00 (11.00, 20.00) | 15.00 (11.00, 19.00) | Z=-0.60 | 0.549 |
| Body Mass Index (kg/m²) ,M (Q1, Q3) | 24.04 (22.23, 26.35) | 24.07 (22.27, 26.35) | 24.03 (22.13, 26.22) | Z=-0.82 | 0.414 |
| Diastasis Recti (cm) ,M (Q1, Q3) | 2.00 (1.50, 2.50) | 2.00 (1.50, 2.50) | 2.00 (1.50, 2.50) | Z=-0.05 | 0.960 |
| Pelvic Pressure (cmH_2_O) ,M (Q1, Q3) | 63.00 (57.00, 72.00) | 63.00 (57.00, 71.00) | 63.10 (58.00, 73.00) | Z=-1.21 | 0.227 |
| **Education Level(%) ,M (Q1, Q3)** |  |  |  | χ²=0.47 | 0.791 |
| Below High School | 93 (8.73) | 64 (8.59) | 29 (9.06) |  |  |
| Below Bachelor's Degree | 536 (50.33) | 371 (49.80) | 165 (51.56) |  |  |
| Bachelor's Degree or Above | 436 (40.94) | 310 (41.61) | 126 (39.38) |  |  |
| **Economic Level (%)** |  |  |  | χ²=0.70 | 0.705 |
| Poor | 432 (40.56) | 305 (40.94) | 127 (39.69) |  |  |
| Moderate | 516 (48.45) | 362 (48.59) | 154 (48.12) |  |  |
| Good | 117 (10.99) | 78 (10.47) | 39 (12.19) |  |  |
| **Smoke (%)** |  |  |  | χ²=1.33 | 0.248 |
| Yes | 125 (11.74) | 93 (12.48) | 32 (10.00) |  |  |
| No | 940 (88.26) | 652 (87.52) | 288 (90.00) |  |  |
| **Drink (%)** |  |  |  | χ²=0.01 | 0.911 |
| Yes | 78 (7.32) | 55 (7.38) | 23 (7.19) |  |  |
| No | 987 (92.68) | 690 (92.62) | 297 (92.81) |  |  |
| **Preterm Birth (%)** |  |  |  | χ²=4.55 | **0.033** |
| Yes | 73 (6.85) | 43 (5.77) | 30 (9.38) |  |  |
| No | 992 (93.15) | 702 (94.23) | 290 (90.62) |  |  |
| **Number of Births (%)** |  |  |  | χ²=0.12 | 0.731 |
| 1 | 1055 (99.06) | 739 (99.19) | 316 (98.75) |  |  |
| 2 | 10 (0.94) | 6 (0.81) | 4 (1.25) |  |  |
| **Cesarean Section (%)** |  |  |  | χ²=1.69 | 0.193 |
| Yes | 448 (42.07) | 323 (43.36) | 125 (39.06) |  |  |
| No | 617 (57.93) | 422 (56.64) | 195 (60.94) |  |  |
| **Painless Delivery (%)** |  |  |  | χ²=5.44 | **0.020** |
| Yes | 551 (51.74) | 368 (49.40) | 183 (57.19) |  |  |
| No | 514 (48.26) | 377 (50.60) | 137 (42.81) |  |  |
| **Episiotomy (%)** |  |  |  | χ²=2.09 | 0.149 |
| Yes | 192 (18.03) | 126 (16.91) | 66 (20.62) |  |  |
| No | 873 (81.97) | 619 (83.09) | 254 (79.38) |  |  |
| **Perineal Laceration (%)** |  |  |  | χ²=1.44 | 0.230 |
| Yes | 453 (42.54) | 308 (41.34) | 145 (45.31) |  |  |
| No | 612 (57.46) | 437 (58.66) | 175 (54.69) |  |  |
| **Forceps Delivery (%)** |  |  |  | χ²=4.44 | **0.035** |
| Yes | 32 (3.00) | 17 (2.28) | 15 (4.69) |  |  |
| No | 1033 (97.00) | 728 (97.72) | 305 (95.31) |  |  |
| **Manual Removal of Placenta (%)** |  |  |  | χ²=1.08 | 0.299 |
| Yes | 158 (14.84) | 105 (14.09) | 53 (16.56) |  |  |
| No | 907 (85.16) | 640 (85.91) | 267 (83.44) |  |  |
| **Vaginal Bleeding (%)** |  |  |  | χ²=0.46 | 0.496 |
| Yes | 216 (20.28) | 147 (19.73) | 69 (21.56) |  |  |
| No | 849 (79.72) | 598 (80.27) | 251 (78.44) |  |  |
| **Postpartum Pain (%)** |  |  |  | χ²=0.76 | 0.384 |
| Yes | 222 (20.85) | 150 (20.13) | 72 (22.50) |  |  |
| No | 843 (79.15) | 595 (79.87) | 248 (77.50) |  |  |
| **Urinary Dysfunction (%)** |  |  |  | χ²=3.43 | 0.064 |
| Yes | 157 (14.74) | 100 (13.42) | 57 (17.81) |  |  |
| No | 908 (85.26) | 645 (86.58) | 263 (82.19) |  |  |
| **Bowel Dysfunction (%)** |  |  |  | χ²=1.27 | 0.259 |
| Yes | 166 (15.59) | 110 (14.77) | 56 (17.50) |  |  |
| No | 899 (84.41) | 635 (85.23) | 264 (82.50) |  |  |
| **Thyroid Abnormalities During Pregnancy (%)** |  |  |  | χ²=0.87 | 0.351 |
| Yes | 44 (4.13) | 28 (3.76) | 16 (5.00) |  |  |
| No | 1021 (95.87) | 717 (96.24) | 304 (95.00) |  |  |
| **Pregnancy Induced Hypertension (%)** |  |  |  | χ²=0.50 | 0.482 |
| Yes | 29 (2.72) | 22 (2.95) | 7 (2.19) |  |  |
| No | 1036 (97.28) | 723 (97.05) | 313 (97.81) |  |  |
| **Gestational Diabetes (%)** |  |  |  | χ²=4.21 | **0.040** |
| Yes | 169 (15.87) | 107 (14.36) | 62 (19.38) |  |  |
| No | 896 (84.13) | 638 (85.64) | 258 (80.62) |  |  |
| **Pregnancy Complications (%)** |  |  |  | χ²=3.84 | 0.050 |
| Yes | 242 (22.72) | 157 (21.07) | 85 (26.56) |  |  |
| No | 823 (77.28) | 588 (78.93) | 235 (73.44) |  |  |
| **Fetal Weight Abnormality (%)** |  |  |  | χ²=1.49 | 0.222 |
| Yes | 409 (38.40) | 295 (39.60) | 114 (35.62) |  |  |
| No | 656 (61.60) | 450 (60.40) | 206 (64.38) |  |  |
| **Pre-pregnancy menstrual cycle abnormalities (%)** |  |  |  | χ²=0.02 | 0.893 |
| Yes | 132 (12.39) | 93 (12.48) | 39 (12.19) |  |  |
| No | 933 (87.61) | 652 (87.52) | 281 (87.81) |  |  |
| **Primipara (%)** |  |  |  | χ²=0.42 | 0.515 |
| Yes | 866 (81.31) | 602 (80.81) | 264 (82.50) |  |  |
| No | 199 (18.69) | 143 (19.19) | 56 (17.50) |  |  |
| **Adverse Obstetric History (%)** |  |  |  | χ²=0.06 | 0.802 |
| Yes | 337 (31.64) | 234 (31.41) | 103 (32.19) |  |  |
| No | 728 (68.36) | 511 (68.59) | 217 (67.81) |  |  |
| **Adequate Breast Milk(%)** |  |  |  | χ²=0.58 | 0.445 |
| Yes | 684 (64.23) | 473 (63.49) | 211 (65.94) |  |  |
| No | 381 (35.77) | 272 (36.51) | 109 (34.06) |  |  |
| **Feeding Method (%)** |  |  |  | χ²=1.41 | 0.235 |
| Breastfeeding | 897 (84.23) | 621 (83.36) | 276 (86.25) |  |  |
| Mixed Feeding | 168 (15.77) | 124 (16.64) | 44 (13.75) |  |  |
| **Abdominal Scar (%)** |  |  |  | χ²=0.43 | 0.512 |
| Yes | 422 (39.62) | 300 (40.27) | 122 (38.12) |  |  |
| No | 643 (60.38) | 445 (59.73) | 198 (61.88) |  |  |
| **Pubic Symphysis Pain (%)** |  |  |  | χ²=3.09 | 0.079 |
| Yes | 265 (24.88) | 174 (23.36) | 91 (28.44) |  |  |
| No | 800 (75.12) | 571 (76.64) | 229 (71.56) |  |  |
| **Vulva (%)** |  |  |  | χ²=0.78 | 0.378 |
| Normal | 441 (41.41) | 302 (40.54) | 139 (43.44) |  |  |
| Abnormal | 624 (58.59) | 443 (59.46) | 181 (56.56) |  |  |
| **Vagina (%)** |  |  |  | χ²=0.03 | 0.857 |
| Normal | 835 (78.40) | 583 (78.26) | 252 (78.75) |  |  |
| Abnormal | 230 (21.60) | 162 (21.74) | 68 (21.25) |  |  |
| **Cervix (%)** |  |  |  | χ²=0.00 | 0.965 |
| Normal | 916 (86.01) | 641 (86.04) | 275 (85.94) |  |  |
| Abnormal | 149 (13.99) | 104 (13.96) | 45 (14.06) |  |  |
| **Uterus (%)** |  |  |  | χ²=0.35 | 0.555 |
| Normal | 1037 (97.37) | 724 (97.18) | 313 (97.81) |  |  |
| Abnormal | 28 (2.63) | 21 (2.82) | 7 (2.19) |  |  |
| **Adnexa (%)** |  |  |  | χ²=0.06 | 0.810 |
| Normal | 1033 (97.00) | 722 (96.91) | 311 (97.19) |  |  |
| Abnormal | 32 (3.00) | 23 (3.09) | 9 (2.81) |  |  |
| **Hemorrhoids (%)** |  |  |  | χ²=0.18 | 0.674 |
| Yes | 453 (42.54) | 320 (42.95) | 133 (41.56) |  |  |
| No | 612 (57.46) | 425 (57.05) | 187 (58.44) |  |  |
| **Pelvic Floor Tenderness (%)** |  |  |  | χ²=0.44 | 0.508 |
| Yes | 89 (8.36) | 65 (8.72) | 24 (7.50) |  |  |
| No | 976 (91.64) | 680 (91.28) | 296 (92.50) |  |  |
| **Fetal Sex Preference (%)** |  |  |  | χ²=0.01 | 0.908 |
| Yes | 287 (26.95) | 200 (26.85) | 87 (27.19) |  |  |
| No | 778 (73.05) | 545 (73.15) | 233 (72.81) |  |  |
| **Planned Pregnancy (%)** |  |  |  | χ²=0.56 | 0.453 |
| Yes | 745 (69.95) | 516 (69.26) | 229 (71.56) |  |  |
| No | 320 (30.05) | 229 (30.74) | 91 (28.44) |  |  |
| **Prenatal Education Class (%)** |  |  |  | χ²=0.40 | 0.525 |
| Yes | 404 (37.93) | 278 (37.32) | 126 (39.38) |  |  |
| No | 661 (62.07) | 467 (62.68) | 194 (60.62) |  |  |
| **Perinatal Sleep Status (%)** |  |  |  | χ²=5.76 | 0.056 |
| Good | 626 (58.78) | 454 (60.94) | 172 (53.75) |  |  |
| Average | 268 (25.16) | 173 (23.22) | 95 (29.69) |  |  |
| Poor | 171 (16.06) | 118 (15.84) | 53 (16.56) |  |  |
| **Prenatal Anxiety (%)** |  |  |  | χ²=0.13 | 0.715 |
| Yes | 217 (20.38) | 154 (20.67) | 63 (19.69) |  |  |
| No | 848 (79.62) | 591 (79.33) | 257 (80.31) |  |  |
| **Satisfaction With Postpartum Confinement (%)** |  |  |  | χ²=2.64 | 0.105 |
| Satisfied | 762 (71.55) | 544 (73.02) | 218 (68.12) |  |  |
| Unsatisfied | 303 (28.45) | 201 (26.98) | 102 (31.88) |  |  |
| **Marital Relationship (%)** |  |  |  | χ²=3.05 | 0.081 |
| Good | 955 (89.67) | 676 (90.74) | 279 (87.19) |  |  |
| Poor | 110 (10.33) | 69 (9.26) | 41 (12.81) |  |  |
| **In law Relationship (%)** |  |  |  | χ²=0.14 | 0.710 |
| Good | 879 (82.54) | 617 (82.82) | 262 (81.88) |  |  |
| Poor | 186 (17.46) | 128 (17.18) | 58 (18.12) |  |  |
| **Pelvic Floor Muscle Strength (%)** |  |  |  | χ²=1.38 | 0.926 |
| 0 | 22 (2.07) | 15 (2.01) | 7 (2.19) |  |  |
| 1 | 381 (35.77) | 270 (36.24) | 111 (34.69) |  |  |
| 2 | 355 (33.33) | 245 (32.89) | 110 (34.38) |  |  |
| 3 | 206 (19.34) | 145 (19.46) | 61 (19.06) |  |  |
| 4 | 78 (7.32) | 56 (7.52) | 22 (6.88) |  |  |
| 5 | 23 (2.16) | 14 (1.88) | 9 (2.81) |  |  |
| **Pelvic Floor Muscle Endurance (%)** |  |  |  | χ²=7.92 | 0.161 |
| 0 | 78 (7.32) | 61 (8.19) | 17 (5.31) |  |  |
| 1 | 498 (46.76) | 341 (45.77) | 157 (49.06) |  |  |
| 2 | 285 (26.76) | 198 (26.58) | 87 (27.19) |  |  |
| 3 | 135 (12.68) | 93 (12.48) | 42 (13.12) |  |  |
| 4 | 50 (4.69) | 41 (5.50) | 9 (2.81) |  |  |
| 5 | 19 (1.78) | 11 (1.48) | 8 (2.50) |  |  |
| **Type I Pelvic Floor Muscles (%)** |  |  |  | χ²=2.64 | 0.756 |
| 0 | 315 (29.58) | 220 (29.53) | 95 (29.69) |  |  |
| 1 | 273 (25.63) | 191 (25.64) | 82 (25.62) |  |  |
| 2 | 131 (12.30) | 85 (11.41) | 46 (14.38) |  |  |
| 3 | 80 (7.51) | 57 (7.65) | 23 (7.19) |  |  |
| 4 | 34 (3.19) | 26 (3.49) | 8 (2.50) |  |  |
| 5 | 232 (21.78) | 166 (22.28) | 66 (20.62) |  |  |
| **Type II Pelvic Floor Muscles (%)** |  |  |  | χ²=2.13 | 0.830 |
| 0 | 212 (19.91) | 156 (20.94) | 56 (17.50) |  |  |
| 1 | 144 (13.52) | 98 (13.15) | 46 (14.38) |  |  |
| 2 | 123 (11.55) | 83 (11.14) | 40 (12.50) |  |  |
| 3 | 99 (9.30) | 69 (9.26) | 30 (9.38) |  |  |
| 4 | 74 (6.95) | 53 (7.11) | 21 (6.56) |  |  |
| 5 | 413 (38.78) | 286 (38.39) | 127 (39.69) |  |  |
| Postpartum Depression, n(%) |  |  |  | χ²=0.17 | 0.684 |
| No | 814 (76.43) | 572 (76.78) | 242 (75.62) |  |  |
| Yes | 251 (23.57) | 173 (23.22) | 78 (24.38) |  |  |
| Z: Mann-Whitney test, χ²: Chi-square test |  |  |  |  |  |
| M: Median, Q₁: 1st Quartile, Q₃: 3st Quartile |  |  |  |  |  |
